# Free Medial Meniscal Fragment Which Mimics the Dislocated Bucket-Handle Tear on MRI

**DOI:** 10.1155/2014/647491

**Published:** 2014-06-09

**Authors:** Faik Türkmen, İsmail Hakkı Korucu, Cem Sever, Mehmet Demirayak, Gani Goncü, Serdar Toker

**Affiliations:** ^1^Department of Orthopedics, NEU Meram School of Medicine, Akyokus, Meram, Konya, Turkey; ^2^Department of Orthopedics, Mevlana University School of Medicine, Konya, Turkey

## Abstract

The bucket-handle meniscal tear is a specific type of meniscal injuries which has specific signs on MRI. An attached fragment displaced away from the meniscus with any type of tear causes bucket-handle tear of the meniscus. Magnetic resonance imaging (MRI) is the most commonly used diagnostic tool for meniscal injuries. We present a case of free medial meniscal fragment which mimics the dislocated bucket-handle tear on MRI. The presence of “fragment within the intercondylar notch sign” and “the absence of the bow tie sign” may be an indication of a free meniscal fragment. This should be considered during diagnosis.

## 1. Introduction

The menisci are semilunar structures which are localized in the knee joint. Numerous functions of the menisci have been described until now. Meniscal injuries may occur due to trauma, degeneration, or both.

Meniscal tears are generally classified according to whether being vertical, horizontal, or complex [1]. The bucket-handle meniscal tear is a specific type of meniscal injuries which has specific signs on MRI. An attached fragment displaced away from the meniscus with any type of tear causes bucket-handle tear of the meniscus [2].

Magnetic resonance imaging (MRI) is the most commonly used diagnostic tool for meniscal injuries. The accuracy of MRI for meniscal imaging ranges from 45 to 98% according to several studies [3–8]. An abnormal signal within the meniscus and abnormalities in meniscal morphology are the general signs of meniscal tears.

We present a case of free medial meniscal fragment which mimics the dislocated bucket-handle tear on MRI.

## 2. Case Report

**Figure 1:**
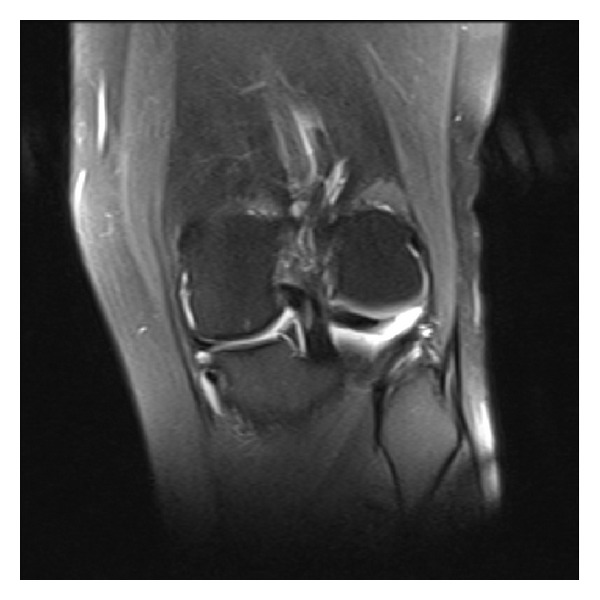
Coronal MR image demonstrates decreased volume of the medial meniscus and a meniscal fragment within the intercondylar notch.

**Figure 2:**
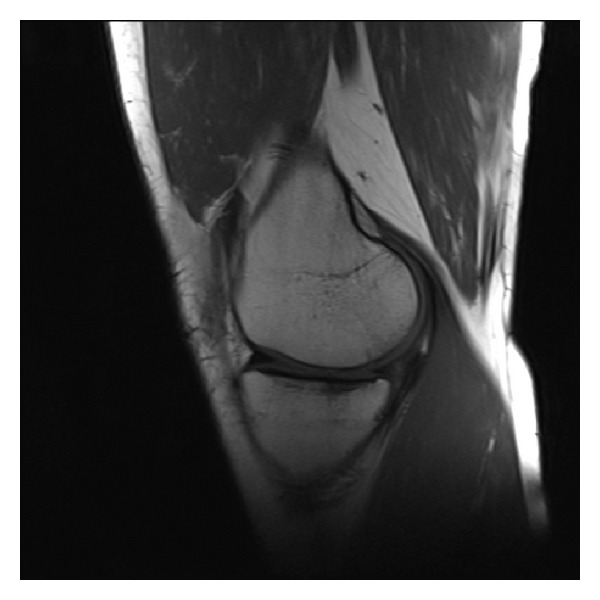
Sagittal MR image demonstrates “absence of the bow tie sign.”

A 29-year-old man had a left knee injury during an amateur football match 2 years ago. He had been diagnosed as having a tear of the medial meniscus and received a meniscus repair under arthroscopy at another medical center. He had a left knee injury similar to the first one 14 months after the first operation. He visited our clinic 6 months after the second injury. He had complained of left knee pain and anterior instability. On physical examination he had slight tenderness and anterior cruciate ligament (ACL) derangement on his left knee. Anterior drawer, Lachman's, and pivot shift tests were positive on physical examination. A complete rupture of the ACL and signs of a bucket-handle tear of the medial meniscus were detected on MRI. Medial meniscus volume was found to be decreased at the medial compartment and a meniscal fragment within the intercondylar notch was detected in the coronal view (Figure 1). There was also “absence of the bow tie sign” in the sagittal view (Figure 2). Arthroscopic medial meniscal repair and ACL reconstruction were planned. The patient underwent arthroscopic surgery. Arthroscopy demonstrated that a completely free large meniscal fragment which has not any connection with the rest of the meniscus was tightly connected to the posterior cruciate ligament (PCL). The volume of the free fragment was approximately 60–65% of the entire volume of the medial meniscus. It was released and excised arthroscopically. There was not any tear or other visible pathology in the rest of the meniscus. ACL reconstruction was performed in the same session.

## 3. Discussion

The menisci are semilunar structures located within the knee joint. There are many functions of the menisci such as distribution of the load over the articular cartilage, shock absorption, joint lubrication, and stabilization of the knee [1, 9].

Meniscal injuries may occur due to trauma, degeneration, or both. An increased force may cause a tear on the normal meniscus; however, a degenerative meniscus may be injured due to normal forces.

Magnetic resonance imaging (MRI) is the most commonly used diagnostic tool for meniscal injuries. An increased internal signal and meniscal shape abnormality indicate meniscal tears on MR images. Meniscal tears can be described as vertical, horizontal, or complex [1]. Bucket-handle meniscal tear is a longitudinal, vertical, or oblique tear with an attached fragment displaced away from the meniscus [10]. The fragment of medial meniscus may be displaced into the intercondylar notch in a bucket-handle tear. Well-known MRI findings of bucket-handle meniscal tears are “fragment within the intercondylar notch sign,” “the absence of the bow tie sign,” “double PCL sign,” and “anterior flipped meniscus sign.” Other findings are “double anterior horn sign,” “disproportional posterior horn sign,” and “coronal truncation sign” [11, 12].

Medial meniscus volume was found to be decreased at the medial compartment and a meniscal fragment within the intercondylar notch was detected in the coronal view of MRI in our case. There was also “absence of the bow tie sign” in the sagittal view. These findings were thought to be due to the dislocated bucket-handle tear of the medial meniscus. However, arthroscopy demonstrated that a completely free large meniscal fragment which has not any connection with the rest of the meniscus caused these findings on MRI.

The preferred treatment of bucket-handle meniscal tears is arthroscopic repair of the displaced fragment if possible. Arthroscopic resection is performed for irreparable meniscal fragments. The free meniscal fragment which was tightly connected to the PCL was released and excised arthroscopically in our case.

The finding of this case report is that a free meniscal fragment can mimic the dislocated bucket-handle tear on MRI. “Fragment within the intercondylar notch sign” and “the absence of the bow tie sign” may be due to a free meniscal fragment which has not any connection to the meniscus.
